# Comparison of environmental inference approaches for ecometric analyses: Using hypsodonty to estimate precipitation

**DOI:** 10.1002/ece3.7081

**Published:** 2020-12-04

**Authors:** Rachel A. Short, Katherine Pinson, A. Michelle Lawing

**Affiliations:** ^1^ Department of Ecology and Conservation Biology Texas A&M University College Station TX USA; ^2^ Department of Geology and Geophysics Texas A&M University College Station TX USA

**Keywords:** ecometrics, hypsodonty, precipitation, trait–environment relationships

## Abstract

1. Ecometrics is the study of community‐level functional trait–environment relationships. We use ecometric analyses to estimate paleoenvironment and to investigate community‐level functional changes through time.
2. We evaluate four methods that have been used or have the potential to be used in ecometric analyses for estimating paleoenvironment to determine whether there have been systematic differences in paleoenvironmental estimation due to choice of the estimation method. Specifically, we evaluated linear regression, polynomial regression, nearest neighbor, and maximum‐likelihood methods to explore the predictive ability of the relationship for a well‐known ecometric dataset of mammalian herbivore hypsodonty metrics (molar tooth crown to root height ratio) and annual precipitation. Each method was applied to 43 Pleistocene fossil sites and compared to annual precipitation from global climate models. Sites were categorized as glacial or interglacial, and paleoprecipitation estimates were compared to the appropriate model.
3. Estimation methods produce results that are highly correlated with log precipitation and estimates from the other methods (*p* < 0.001). Differences between estimated precipitation and observed precipitation are not significantly different across the four methods, but maximum likelihood produces the most accurate estimates of precipitation. When applied to paleontological sites, paleoprecipitation estimates align more closely with glacial global climate models than with interglacial models regardless of the age of the site.
4. Each method has constraints that are important to consider when designing ecometric analyses to avoid misinterpretations when ecometric relationships are applied to the paleontological record. We show interglacial fauna estimates of paleoprecipitation more closely match glacial global climate models. This is likely because of the anthropogenic effects on community reassembly in the Holocene.

## Graphical abstract

We evaluate four statistical methods that have been used or have the potential to be used in ecometric analyses for estimating paleoenvironment to determine whether there have been systematic differences in paleoenvironmental estimation due to choice of the estimation method. We show that differences between estimated precipitation and observed precipitation are not significantly different across the four methods, but maximum likelihood produces the most accurate estimates of precipitation. When applied to paleontological sites, paleoprecipitation estimates align more closely with glacial global climate models than with interglacial models regardless of the age of the site.

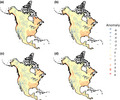

## 1 INTRODUCTION

Functional traits are measurable features that influence an organism's interaction with its environment (McGill et al., 2006; Violle et al., 2007). When measured in the fossil record, functional traits can be used for a thorough understanding of biotic responses to corresponding environmental changes (Eronen, Polly, et al., 2010), which can contribute to improved predictions of future faunal communities as they face severe impacts from environmental change (Barnosky et al., 2011; Ceballos et al., 2005, 2015). With climate expected to continue changing at unprecedented rates (Intergovernmental Panel on Climate Change, 2014; Wuebbles et al., 2017), it is important to better understand the past so that we can anticipate future faunal responses.

Ecometric analyses were developed to estimate paleoclimatic conditions from fossil assemblages by providing a linkage between paleontological data, modern data, and projections of functional responses to impending climate change (Polly et al., 2011; Polly & Head, 2015). These studies use the trait–environment relationship to study assemblage‐level responses over spatial and temporal scales (Eronen, Polly, et al., 2010; Polly et al., 2011; Polly & Head, 2015). When there is a strong trait–environment relationship, the traits can act as predictors of environment (Eronen, Polly, et al., 2010; McGill et al., 2006), and paleontology can inform conservation efforts by providing a long‐term record of change (Barnosky et al., 2017; Dietl & Flessa, 2011; Dietl et al., 2015).

Previous research has demonstrated relationships between community‐level trait composition and environmental variables, including for plant leaf margins (Nicotra et al., 2011; Peppe et al., 2011; Royer et al., 2012; Wolfe, 1979), herbivore teeth (Eronen, Polly, et al., 2010; Eronen et al., 2010a; Evans, 2013; Fortelius et al., 2016), and locomotor skeletal elements of bovids (Barr, 2017), carnivorans (Polly, 2010), and snakes (Lawing et al., 2012), but the estimation methods have varied. Wolfe (1979) used linear regression to demonstrate that areas with high mean annual temperatures are dominated by leaves with entire margins while areas with low temperatures are dominated by leaves with nonentire margins. Eronen et al. (2010b) used linear regression and regression tree analysis to estimate Eurasian paleoprecipitation from large mammal hypsodonty values. Barr (2017) used general linear models to study the relationship between bovid postcranial elements and vegetation cover and precipitation. Fortelius et al. (2016) used regression and k‐nearest neighbor (kNN) analyses on dental characters to investigate paleoenvironment in the Turkana Basin between 7 and 1 million years ago. Polly (2010) and Lawing et al. (2012) used maximum‐likelihood estimation to explore the ecometric value of carnivoran calcaneal morphology and relative snake tail length, respectively. The community of scientists using ecometrics for conservation paleontology will benefit from a discussion of when to use which methods because less accurate methods will cause misinterpretations when ecometric relationships are applied to the paleontological record.

Although the use of ecometrics has increased in recent years, only Fortelius et al. (2016) compare multiple methods—regression and k‐nearest neighbor (kNN)—by also using hypsodonty as the ecometric trait. In this case, the authors discuss merits of both including that regression is easier to interpret because it produces an equation and that kNN is more sensitive to variation because it is nonlinear. An analysis of additional estimation methods will enable better comparisons and address potential weaknesses of paleoenvironmental interpretations.

### 1.1 Herbivore hypsodonty

Hypsodonty is the ratio of the tooth crown height to root height of the molars, and the relationship between hypsodonty and annual precipitation and is highly correlated in large and small mammals (Eronen, Polly, et al., 2010; Eronen et al., 2010a; Lawing et al., 2017). Hypsodonty is functionally related to the durability of teeth in herbivores and provides biomechanical advantages, including more restricted areas of stress and increased occlusal pressure, to support more efficient mastication of grass and other tough, poor quality vegetation (Demiguel et al., 2016; Solounias et al., 2019). Increased hypsodonty has been linked to more roughage in the diet (Erickson, 2014; Merceron et al., 2016; Strömberg, 2002, 2006) and increased environmental grit consumed during feeding (Damuth & Janis, 2011; Jardine et al., 2012; Semprebon et al., 2019). In small mammals, it is common for taxa who do not eat grass to have hypsodont dentition and to inhabit arid environments (Nowak, 1999). With increasing aridity, increasing dietary roughage and increasing environmental grit often coincide, so that both diet and habitat play a role in the development of hypsodont dentition (Fortelius et al., 2002; Toljagić et al., 2018; Williams & Kay, 2001). Therefore, as environments have changed, so too have community‐level hypsodonty values.

Records from the Great Plains and the western United States suggest that North American habitats became more open and grass‐dominated in the Miocene (Edwards et al., 2010; Strömberg, 2011). There were approximately 4 million years between the establishment of C_3_ grasslands and the origination of equid hypsodonty in the Great Plains of North America (Strömberg, 2006); it was approximately another 10 million years until specialized grazing ungulates appeared (Janis, 2008). However, rodents and lagomorphs responded millions of years earlier than the ungulates (Samuels & Hopkins, 2017).

Eventually, there was a turnover from predominately low‐crowned to high‐crowned taxa, so that large mammal communities with higher hypsodonty indices are generally found in more open and arid grasslands (Eronen, Polly, et al., 2010; Eronen et al., 2010b; Fortelius et al., 2002; Janis et al., 2000, 2002; Strömberg, 2011). Annual precipitation estimates based on tooth morphology closely match estimates from climate modeling and paleovegetation records in Eurasia over the past 23 million years (Eronen, Polly, et al., 2010; Eronen et al., 2010b), and the same trait–environment relationship has been used to indicate changes in precipitation in Eurasia (Eronen et al., 2012; Fortelius et al., 2002), Italy (Meloro & Kovarovic, 2013), and Kenya (Žliobaitė et al., 2016).

Here, we use the trait–environment relationship between hypsodonty and annual precipitation to compare four methods of ecometric estimation – linear regression, polynomial regression, nearest neighbor, and maximum likelihood. We aim to (a) explore differences in the predictive ability of each method and (b) apply each method to Late Pleistocene fossil localities to demonstrate the potential impact of method selection on paleoenvironmental interpretations. We expect maximum likelihood to produce the most accurate estimates of precipitation from community hypsodonty values because the method estimates precipitation by fitting a model to a localized subset of communities that have similar trait values. For that reason, we also expect maximum likelihood to estimate paleoprecipitation that most closely align with global climate models.

## 2 MATERIALS AND METHODS

We used modern communities of herbivores and annual precipitation data to evaluate four estimation methods for ecometric analyses and investigate the capacity of each method to estimate paleoprecipitation for paleontological sites.

### 2.1 Study area and taxa

We use the extant species of Artiodactyla, Perissodactyla, Rodentia, and Lagomorpha (*n* = 404) in North America, because they represent the primary herbivores in North American mammalian communities. Jardine et al. (2012) suggested not including fossorial rodents and lagomorphs in studies of precipitation because these taxa are under selective pressures that do not covary with aridity. However, hypsodonty, as well as fossorial behavior, of small mammals increased as habitats became more dry and open (Samuels & Hopkins, 2017; Schap et al., 2020), and the relationship between hypsodonty and precipitation occurs in Dipodidae, which includes fossorial species outside of North America (Ma et al., 2017). Thus, we have included all Glires here to encompass the majority of the herbivorous mammal community.

We recognize that the North American fauna is biased following the Pleistocene mass extinction (Barnosky et al., 2011; Carrasco et al., 2009) and, therefore, the predictive abilities of the estimation methods may be lower. Megaherbivores, though geographically widespread, do contribute to the community‐level trait values at Pleistocene sites and are not represented in the modern data. However, because the relationship between hypsodonty and precipitation is well‐established, it provides a good dataset for relative comparisons of paleoenvironmental reconstruction methods.

### 2.2 Traits and communities

**FIGURE 1:**
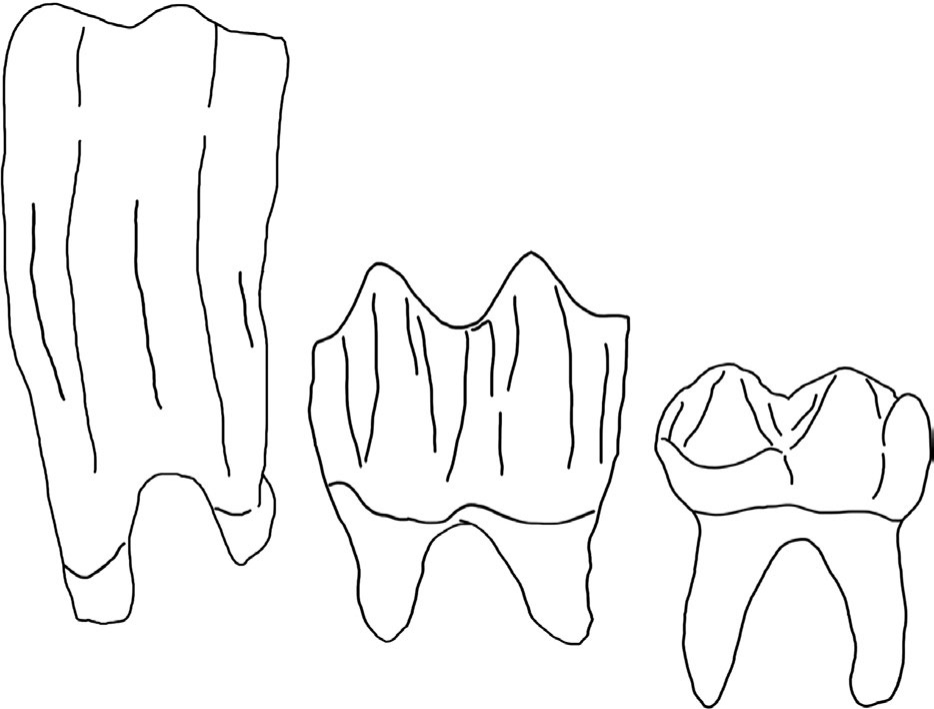
Three levels of hypsodonty examined here. Left, Hypsodont, or high tooth crown–root ratio, as represented by *Equus caballus*; Middle, Mesodont, or moderate tooth crown–root ratio, as represented by *Cervus canadensis*; Right, Brachydont, or low tooth crown–root ratio, as represented by *Tapirus terrestris*

Hypsodonty data for this paper came from an existing dataset, which has been used to investigate trait composition at the community level in North America (Lawing et al., 2017). Crown height for each species was assigned a value of 3 (hypsodont, high crown height), 2 (mesodont, moderate crown height), or 1 (brachydont, low crown height) (Fortelius et al., 2002; Figure 1). An additional 72 species were assigned hypsodonty values based on literature for a total of 446 species. Some members of Rodentia and Lagomorpha have evolved hypselodont dentition in which the teeth continue to emerge throughout the lifespan; these taxa are classified as hypsodont for the purposes of this study following Fortelius et al. (2003).

**FIGURE 2:**
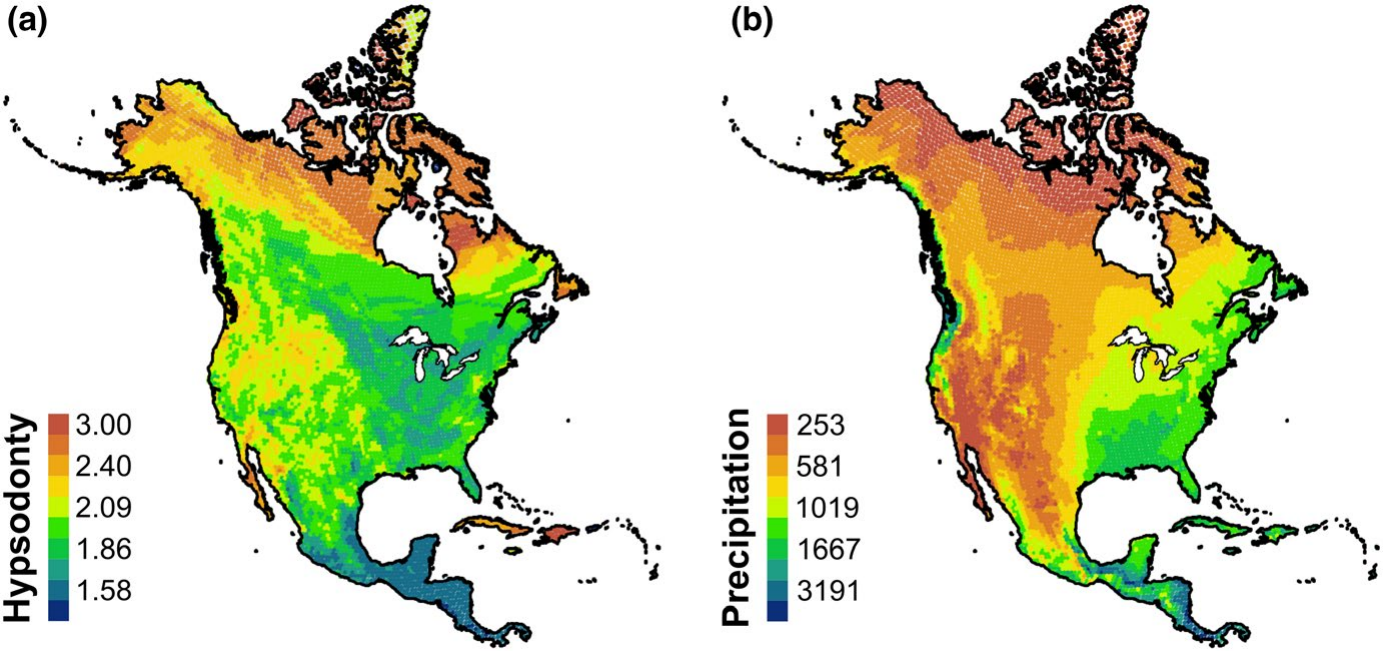
Data used in this study. Legend values are the maximum values for the bin. (a) Mean community hypsodonty values; (b) mean annual precipitation in log mm

Community composition was sampled using an equidistant 50‐km point system (9,699 sampling points) in North America (Lawing et al., 2012, 2017; Polly, 2010) from overlapping expert drawn polygon maps from NatureServe to produce community lists of North American Artiodactyla, Perissodactyla, Rodentia, and Lagomorpha with extant presence and native or reintroduced origin (those data were produced in collaboration with Bruce Patterson, Wes Sechrest, Marcelo Tognelli, Gerardo Ceballos, The Nature Conservancy—Migratory Bird Program, Conservation International—CABS, World Wildlife Fund—US, and Environment Canada—WILDSPACE; Patterson et al., 2007). Taxonomy associated with hypsodonty data and range maps were reviewed to insure consistency following Wilson and Reeder (2005). Only sampling points with a species richness of five or more were kept. Although this may exclude certain communities, it allows for more robust estimates of community‐level measures in our models and enables more rigorous comparisons across estimation methods. We calculated the mean (Figure 2a) and standard deviation of hypsodonty for every sample point.

Our dataset on communities includes the presumed presence or absence of species at each sampling location across North America because the ranges are not based only on direct observations. Another measure of community composition could include recording the presumed abundance of species within communities. That would allow us to weigh the traits by the most commonly occurring taxa (sensu Faith et al., 2019). Faith et al. (2019) show that using abundance instead of occurrence allows for weighted ecometric means that can produce more robust paleoclimate estimates. Despite these benefits, we chose to use occurrences rather than abundance to (a) use range maps in place of observational data for the modern communities, insuring larger coverage, (b) mirror available data at fossil sites that lack abundance descriptions, (c) overcome potential sampling bias that occurs in a dataset that includes both small and large mammals, and (d) replicate methods most commonly used in ecometric studies. In addition, gathering abundance data from the fossil record is highly susceptible to taphonomy and collection practices (Crees et al., 2019; Hernández Fernández & Vrba, 2006).

### 2.3 Environmental data

Annual precipitation data were downloaded from the WorldClim database at the 2.5‐degree grid scale (Hijmans et al., 2005) and extracted at each sampling point across North America (Figure 2b). The natural log of annual precipitation was used to transform the data for normality.

### 2.4 Ecometric analyses

Four inference methods were selected for comparison of mean community hypsodonty to annual precipitation: linear regression, polynomial regression, nearest neighbor, and maximum likelihood. Linear regression and polynomial regression produce estimates using the formula of a line of best fit that is either linear or nonlinear, respectively. Nearest neighbor estimates precipitation by using training data and the *k* closest communities of hypsodonty values. We used 20% of the data as training data and *k* = 15 to include the 15 nearest neighbors in the analysis following Fortelius et al. (2016) who used *k* = 15 using a cross‐validation analysis of hypsodonty and precipitation. For the maximum‐likelihood estimation, communities were binned into 25 × 25 cells based on the mean and standard deviation of their hypsodonty values following Lawing et al. (2012) and Vermillion et al. (2018). Each bin was analyzed to produce the most likely precipitation value for communities with the same trait mean and standard deviation.

Maps of estimated annual precipitation were produced using the community hypsodonty data and each of the inference methods. This estimation step allows for precipitation estimates to be evaluated through comparisons with the observed precipitation dataset. Estimated values were subtracted from the observed values, and differences were mapped to generate anomaly maps (Polly & Sarwar, 2014); smaller differences between estimated and observed values indicate a less biased prediction. Estimates were used to test the Pearson correlation of each method with observed precipitation and the other three methods. An ANOVA test was used to compare the group means across the methods.

### 2.5 Fossil sites application

Inference methods were applied to Late Pleistocene North American fossil sites to demonstrate differences in paleoprecipitation estimates. Case study sites were downloaded from the Paleobiology Database (https://paleobiodb.org) on 12 March 2019, using the following parameters: longitude = −230.449 to 36.5625, latitude = −5.0909 to 64.3969, time interval = Pleistocene, Orders = Artiodactyla, Perissodactyla, Rodentia, and Lagomorpha. Sites were further restricted to the Late Pleistocene (0.126–0.0117 ma) time bin and were limited to communities with at least five species (*n* = 43; Table S1). Fossil taxa were assigned a hypsodonty index value based on literature and the New and Old World (NOW) Database of Fossil Mammals (The NOW Community, 2019). Fossil sites were categorized as interglacial or glacial using literature sources that primarily reported relative dating with many of the site descriptions including either Sangamonian (i.e., interglacial) or Wisconsinan (i.e., glacial) terminology. This final requirement excluded a number of well‐known Pleistocene sites, including Rancho La Brea, American Falls, and Natural Trap Cave, because they could not be easily categorized as either glacial or interglacial.

Global climate models (GCM) were downloaded for the last glacial maximum at 2.5 min resolution (Fick & Hijmans, 2017) and for the last interglacial at 30 arc‐seconds resolution (Fick & Hijmans, 2017; Otto‐Bliesner et al., 2006). Precipitation values were extracted from the GCM models at each site, and an average value was used for the two glacial GCMs—CCSM4 and MIROC‐ESM. These models provided additional precipitation estimates to evaluate the accuracy of the ecometric estimates. For each fossil community, hypsodonty mean and standard deviation were calculated using only one occurrence of each species to prevent duplicating the trait value of any repeated taxa.

Four precipitation estimations were made for each community using the hypsodonty metrics and each inference method. Estimates were compared to the GCM values and to estimates from the other methods using Pearson's correlation tests. Anomalies were calculated by subtracting the estimated values from the GCM values at each site. All analyses were performed in R Statistical Package (R Core Team, 2016).

## 3 RESULTS

**FIGURE 3:**
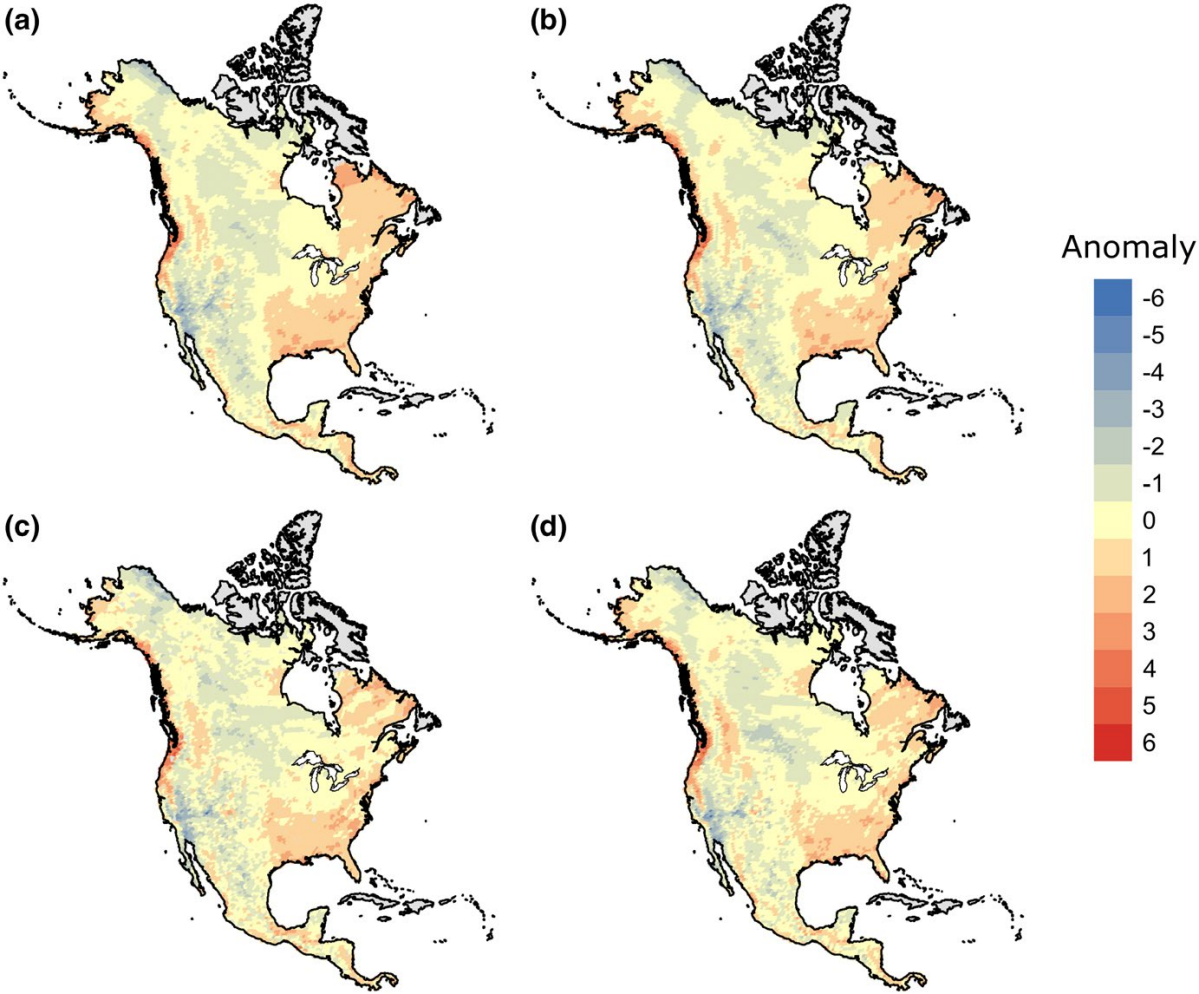
Anomaly maps of differences between observed and estimated precipitation from four estimation methods. (a) linear regression; (b) polynomial regression; (c) nearest neighbor; and (d) maximum likelihood. Scale is log mm and values are the mean of each color bin

Linear regression estimates precipitation with anomalies that range between −4.90 log mm and 4.26 log mm (mean = 0.00 log mm, *y* = −3.34*x* + 12.25, *R* ^2^ = 0.408, *p* < 0.001; Figure 3a). Polynomial regression produces anomalies that range between −4.86 log mm and 4.42 log mm (mean = 0.00 log mm, *y* = 11.95*x* ^3^ + 15.22*x* ^2^ − 73.31*x* + 5.64, *R* ^2^ = 0.436, *p* < 0.001; Figure 3b). These methods overestimate precipitation in dry areas and underestimate precipitation in wet areas. Both regression methods overestimate in the North American deserts, the northern Great Plains, and the tundra and underestimate along the Pacific Northwest coastline, throughout most of the eastern portion of the continent, and somewhat in Central America.

**FIGURE 4:**
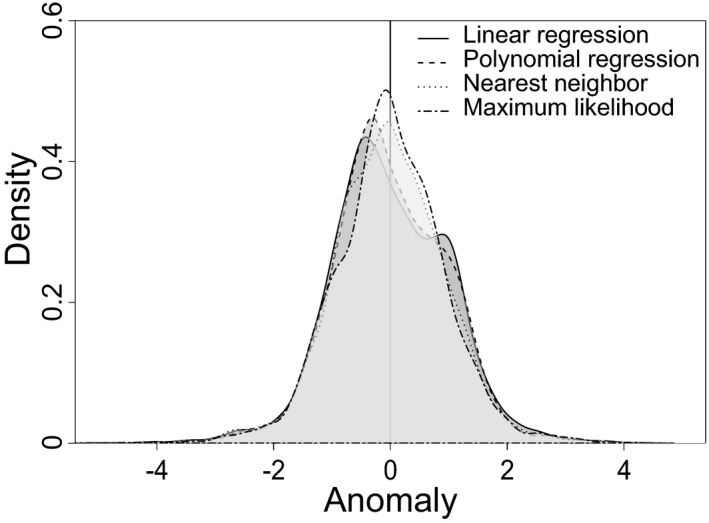
Density of anomalies between observed and estimated precipitation using four estimation methods

Nearest neighbor estimates precipitation anomalies that range between −4.33 log mm and 4.28 log mm (mean = −0.020 log mm; Figure 3c), and maximum likelihood produces anomalies that range between −5.19 log mm and 4.23 log mm (mean = −0.003 log mm; Figure 3d). Nearest neighbor and maximum likelihood overestimate precipitation in dry areas, such as the arid southwest and in the tundra, and underestimate precipitation in wet areas, such as along the Pacific Northwest coast and in the eastern part of the continent. Nearest neighbor also overestimates precipitation in the Rocky Mountains, and maximum likelihood also underestimates precipitation in Central America. There is not a significant difference in anomalies between the four methods (*F*(3, 30,071) = 0.694, *p* = 0.556), but maximum likelihood produces the most neutral (i.e., equal to zero) or nearly neutral anomalies suggesting more accurate estimates overall (Figure 4).

The four estimation methods are all highly correlated with log precipitation and with the other methods, and all correlations are significant at *p* < 0.001 (Table 1). Precipitation is consistently correlated with each of the four methods (*r* = 0.640–0.690). Linear regression and polynomial regression are the most highly correlated methods (*r* = 0.966), whereas linear regression and maximum likelihood are the least correlated methods (*r* = 0.897).

**TABLE 1:** Correlation matrix of observed and estimated precipitation values

	Precipitation (log)	Linear regression	Polynomial regression	Nearest neighbor
Linear regression	0.640			
Polynomial regression	0.663	0.966		
Nearest neighbor	0.673	0.901	0.911	
Maximum likelihood	0.690	0.897	0.930	0.904

Note

All correlations are significant at *p* < 0.001.

### 3.1 Paleoenvironment of fossil sites

**FIGURE 5:**
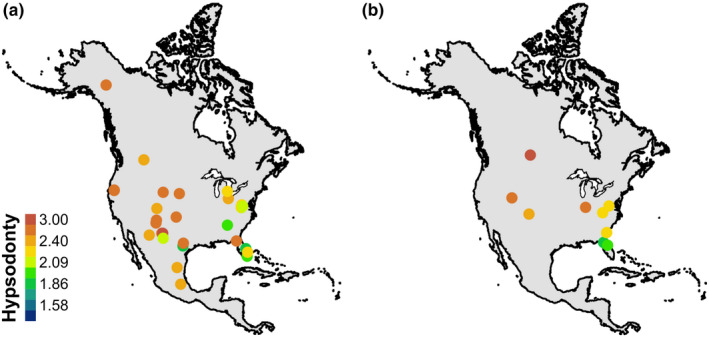
Hypsodonty measures of fossil communities. Hypsodonty values are the maximum values for the bin. (a) Glacial sites; (b) interglacial sites

**FIGURE 6:**
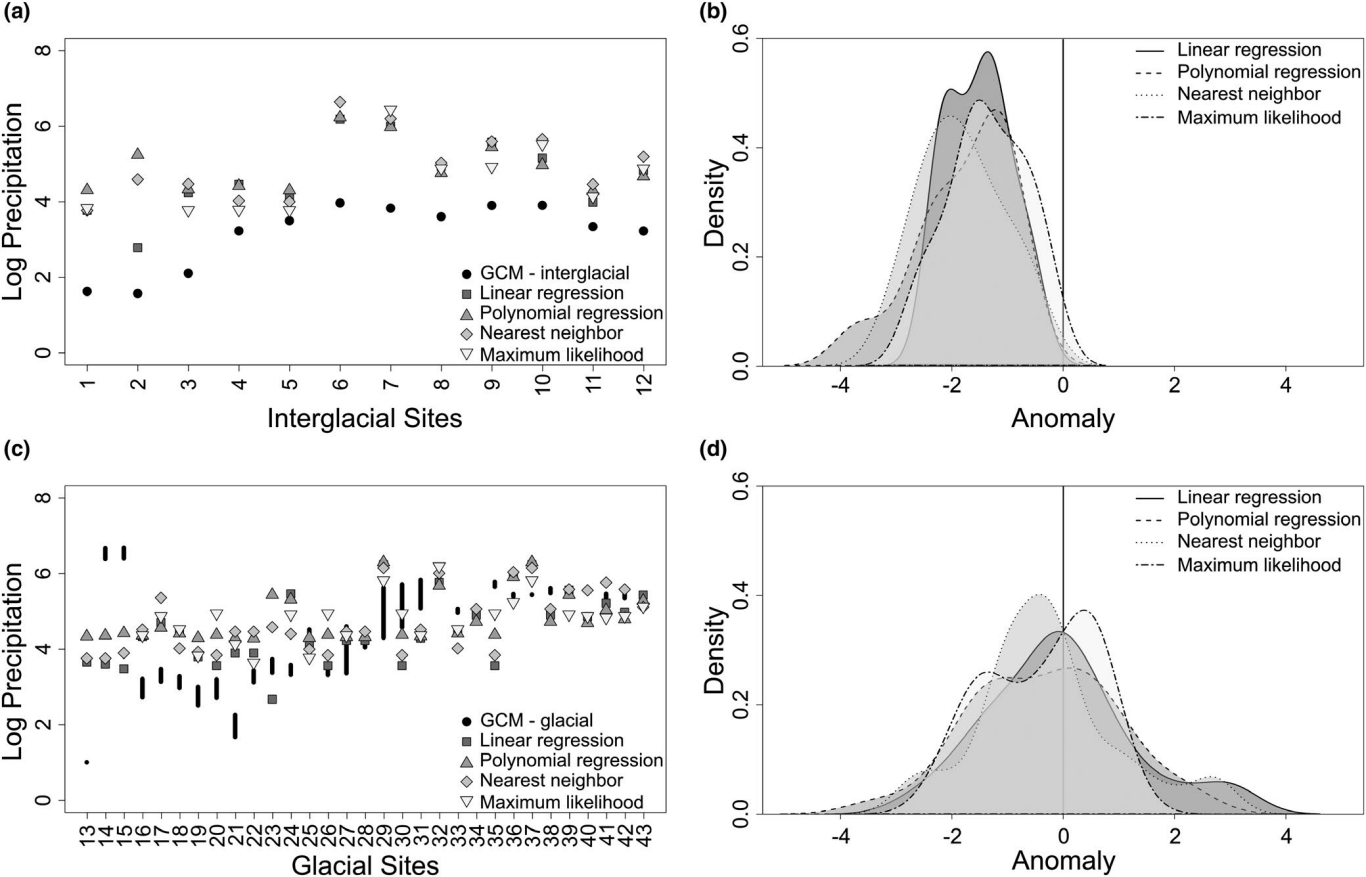
Four estimates of precipitation for glacial and interglacial fossil sites. Glacial and interglacial global climate model estimates are for comparison. (a) Estimates for interglacial sites; (b) density plot of anomalies for interglacial sites; (c) estimates for glacial sites; and (d) density plot of anomalies for glacial sites. Logged precipitation values are provided in Table S1

Most of the paleontological case study sites are glacial (72%; Figure 5). Glacial and interglacial fossil communities are primarily hypsodont with some mesodont communities in the southeast; there are no brachydont communities. Interglacial estimates are higher than the GCMs across all sites (Figure 6a). Interglacial anomalies are centered at approximately −1.5 log mm and have a smaller range than glacial estimates (Figure 6b). At the interglacial sites, maximum likelihood is more closely aligned with the GCM mean. Glacial estimates are higher than the GCMs at high latitudes and converge at approximately 38°N (Strait Canyon, Virginia; Figure 6c). Differences in glacial precipitation estimates and GCMs are centered just below 0 log mm with a small increase at approximately 3 log mm (Figure 6d). Maximum likelihood produces bimodal anomalies at 0 log mm and −2 log mm, but other methods do not display this pattern. Estimates of precipitation at glacial sites more closely match the GCMs than do the estimates at interglacial sites (Figure 6).

## 4 DISCUSSION

Trait–environment relationships can be used for understanding past environmental changes and corresponding biotic responses (Eronen, Polly, et al., 2010; Polly et al., 2011). Because there are minimal differences between estimation methods (Figures 3, 4), we expect that, when a strong ecometric relationship exists, any of the investigated estimation methods will capture the relationship between hypsodonty and precipitation. Therefore, any of the methods can be used to estimate the environment from the distribution of trait values within a community. Hypsodonty and annual precipitation have a well‐established relationship, but these methods may show more differences with a weaker trait–environment relationship.

Each method has constraints that should be considered when selecting a method for ecometric analyses. For example, all estimation methods risk overfitting, but the degree of overfitting depends on the method. Overfitting in a regression analysis depends on the number of parameters; linear regression has a lower risk of overfitting than polynomial regression. The risk of overfitting with maximum likelihood and nearest neighbor largely depends on the size of the bins and neighborhoods, respectively. If the bins or neighborhoods are too small, the model may overfit the data.

Linear regression is the least sensitive to variation in the trait–environment relationship because the estimation model is derived from a fitted regression line. When the model is applied to new trait data to estimate precipitation, each estimate comes from the equation of that regression line. Because precipitation estimates are forced to fit the regression line, there is a reasonable chance of over‐ and underestimation. Therefore, the precipitation estimates from the linear regression model have the weakest correlation with the observed precipitation (Table 1).

Similarly, polynomial regression uses a fitted regression curve of best fit for the estimation model. Estimates of precipitation using polynomial regression place a known hypsodonty value along that curve. In this study, precipitation estimates from polynomial regression are more highly correlated with observed precipitation values than those from linear regression or nearest neighbor (Table 1). However, polynomial regression is unable to predict precipitation values under 4.45 log mm because of the sinusoidal shape of the regression curve. Because of this lower limit of the curve, polynomial regression analyses will overestimate precipitation for communities dominated by taxa with hypsodont dentition because the model cannot estimate low precipitation values. This is particularly relevant for arid regions, such as deserts, that are inhabited by faunal communities with high hypsodonty values.

Nearest neighbor uses a subset of data, that is, training data, to construct a model. A training dataset should be large enough to provide a robust sample for model fit; thus, it is more advantageous to use *k‐*nearest neighbor with a large dataset (Bhatia, 2010). In this study, the training data were 20% of the whole dataset. The *k* value can also be changed to include more or fewer surrounding data points to determine the precipitation value associated with a known reference value. Here, the spatial pattern of overestimation in the arid southwest, tundra, and Rocky Mountains and underestimation in the Pacific Northwest and eastern North America is generally consistent with the other methods (Figure 3c), but precipitation estimates from nearest neighbor have the lowest correlations with the estimates from the other three methods (Table 1).

Maximum likelihood cannot predict precipitation for communities with a trait composition outside of the ecometric trait space used to calibrate the likelihood space. The ecometric trait space is constructed from the trait composition of modern communities. Therefore, in the paleontological case studies, two interglacial sites and seven glacial sites (21% of total sites) did not receive a maximum‐likelihood estimate of precipitation because the hypsodonty values fall outside of the occupied bins designated based on the modern communities (Figure 6). This limitation of the method should be considered when working with potentially nonanalog communities, either in the past or the future, that occur outside of the ecometric trait space. Despite this limitation, precipitation estimates from maximum likelihood are the most highly correlated with observed precipitation (Table 1) and produce the most neutral or nearly neutral anomalies between estimated and observed precipitation values (Figure 4).

Because of evolutionary trends of increasing hypsodont dentition and decreasing brachydont dentition through time (Jardine et al., 2012; Jernvall & Fortelius, 2002; Tapaltsyan et al., 2015), we might expect estimates built on extant taxa to generally underestimate paleoprecipitation. Conversely, because of lags between environmental change and the evolution of hypsodonty (Janis, 2008; Strömberg, 2006), we might expect estimates built on extant taxa to generally overestimate paleoprecipitation. Here, the analyses are on a geologically small temporal scale of approximately 125,000 years, so it is unlikely this evolutionary pattern affected the trait–environment relationship, and the four methods mostly overestimated or accurately predicted precipitation for the fossil sites when compared to the global climate models (Figure 6).

**FIGURE 7:**
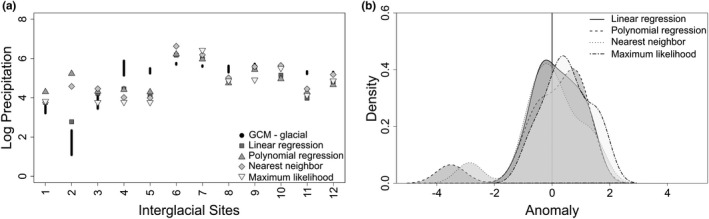
Four estimates of precipitation for interglacial fossil sites compared to glacial global climate model estimates. (a) Estimates for interglacial sites; and (b) density plot of anomalies for interglacial estimates of precipitation and glacial global climate models. Logged precipitation values are provided in Table S1

It might also be expected that today's interglacial fauna should more accurately estimate paleoprecipitation at interglacial sites rather than glacial sites. However, the interglacial estimates are consistently offset from the interglacial global climate models, but more closely align with the glacial global climate models (Figure 7). While this could be an effect of the interglacial precipitation model, it may also be that today's interglacial faunal communities are more similar to the glacial communities. If the extant fauna is lagging behind the climate, the fauna may not have fully responded to today's interglacial conditions. On the timescale of interglacial and glacial cycles, changes in trait composition are driven by community reassembly rather than evolutionary adaptation (Polly et al., 2017). In the Holocene, community assembly is largely affected by anthropogenic effects that have changed community structure patterns to include more segregated species pairs and restricted the interglacial community reassembly that would have occurred without the human impacts (Lyons et al., 2016).

### 4.1 Limitations

In this paper, we used community species lists extracted from expert drawn polygons of species geographic ranges, which typically overestimate species’ presence within communities (Cantú‐Salazar & Gaston, 2013; Jetz et al., 2008). This could affect the trait values of communities that occur along distribution margins and weaken the predictive ability of the estimation methods. Furthermore, although species occurrence data are from distribution estimates updated in 2007 (Patterson et al., 2007), precipitation is an average of data from 1970 to 2000 (Fick & Hijmans, 2017). This temporal mismatch may introduce a bias as faunal assemblages are increasingly affected by anthropogenic pressures, such as land use and habitat loss (Hobbs et al., 2018; Lyons et al., 2016). For example, a current species range map may no longer capture precipitation regime from 1970 to 2000, but may be a reflection of distribution constraints, such as habitat loss and competition from invasive or introduced species.

We have limited our modern community species lists to only native and reintroduced taxa. Extirpation or extinction of native species and the presence of invasives and non‐native species can change the trait values of a community (Žliobaitė et al., 2018), but, with a strong trait–environment relationship, it is unlikely that these taxa would change the trait values enough to notably change the environmental interpretation (Polly & Sarwar, 2014). For instance, it was expected that the Pleistocene megafaunal extinction would create a bias and make the functions unable to estimate precipitation of glacial sites. However, the glacial estimates more closely aligned with the global climate models (Figure 6).

Fossil sites were designated as interglacial and glacial using relative dating. Literature often described the fossil sites as having a Sangamonian (interglacial) or Wisconsinan (glacial) fauna, which made it difficult to use finer temporal resolution. Because of the consistent estimates within interglacial sites and glacial sites (Figure 6), it is unlikely that this caused a misinterpretation of results. It would be beneficial to further evaluate the pattern of overestimating interglacial precipitation across sites using only fossil sites with absolute dating. In general, more studies on fossil communities are needed to increase the applicability of trait‐based models to the past and the future.

### 4.2 Implications

Evaluating ecological and evolutionary processes from data archived in the fossil record provides critical information about biodiversity to researchers, conservationists, and managers by facilitating a better understanding of anticipated biological responses to expected environmental changes (Barnosky et al., 2017; Dietl & Flessa, 2011; Dietl et al., 2015). Paleobiological records provide a broader and deeper perspective that allows us to forecast how impending climate change will affect species and communities (Burney & Burney, 2007; Lawing et al., 2016). Therefore, researchers are increasingly considering conservation implications in their paleontological work and, as such, it is important that we consider the methods used to define the trait–environment relationship. Here, we show that the hypsodonty–precipitation relationship is identifiable with four different estimation methods (Figure 3), although maximum likelihood produces a better fit to observed data and more neutral anomalies than the other methods (Figure 4).

In this study, paleoprecipitation estimates of interglacial fossil communities were more closely aligned with glacial global climate models (Figures 6, 7). This pattern may be due to anthropogenic constraints on community reassembly in the Holocene (Lyons et al., 2016). For instance, today, only 41% of natural areas in the United States demonstrate climate connectivity, so that species can shift their ranges as climate change continues (McGuire et al., 2016). Thus, today's interglacial fauna may not be wholly representative of the fauna from the last interglacial period, but rather is more representative of the last glacial period. Future studies should consider this when working with glacial and interglacial faunal communities.

For a more complete understanding of community responses to environmental change through time, it is imperative that we further explore trait–environment relationships in the paleontological record that can be used in conjunction with other proxies and models, such as global climate models. By using multiple proxies either in parallel or in merged multiproxy models, we can provide a more complete interpretation of past communities, which will be needed to anticipate faunal responses to ongoing environmental changes.

## CONFLICT OF INTEREST

The authors report no conflict of interest.

## AUTHOR CONTRIBUTIONS

**Rachel A. Short:** Conceptualization (equal); data curation (lead); formal analysis (lead); funding acquisition (equal); investigation (lead); methodology (equal); project administration (equal); resources (equal); software (lead); validation (equal); visualization (equal); writing – original draft (lead); writing – review and editing (equal). **Katherine Pinson:** Data curation (supporting); investigation (supporting); visualization (equal); writing – review and editing (equal). **A. Michelle Lawing:** Conceptualization (equal); funding acquisition (equal); methodology (equal); project administration (equal); resources (equal); software (supporting); supervision (lead); validation (equal); visualization (equal); writing – review and editing (equal).

## Supporting information

Table S1

## Data Availability

All data and code are available on the Dryad Digital Repository (https://doi.org/10.5061/dryad.cz8w9gj0t).
